# Transition readiness measures for adolescents with chronic illness: A scoping review of new measures

**DOI:** 10.1016/j.hctj.2023.100022

**Published:** 2023-10-12

**Authors:** Tieghan Killackey, Fareha Nishat, Ellen Elsman, Erica Lawson, Lauren Kelenc, Jennifer N. Stinson

**Affiliations:** a School of Nursing, Faculty of Health, York University, Toronto, Canada; b Child Health Evaluative Sciences, The Hospital for Sick Children, Toronto, Canada; c Department of Pediatrics, University of California, San Francisco, United States; d Institute of Health Policy, Management, and Evaluation, University of Toronto, Canada; e Lawrence S Bloomberg Faculty of Nursing, University of Toronto, Canada

**Keywords:** Transition to adult care, Adolescent chronic illness, Transition readiness measurement

## Abstract

**Background:**

The transition from pediatric to adult care settings for adolescents and young adults living with chronic conditions can be challenging and has been associated with declines in health and access to care. Well-validated measures of patients’ transition readiness are critical, both for use in the clinical setting and to rigorously evaluate transition support programs for the purposes of research and health care quality improvement.

**Objectives:**

This review aimed to build off existing reviews and 1) identify and describe all newly developed and validated measures for the assessment of transition readiness for youth with chronic illness from the period of 2018–2022, and 2) evaluate their measurement properties and identify gaps in measurement testing.

**Methods:**

Electronic searches were conducted in MEDLINE, EMBASE, CINAHL and PsychINFO to identify articles developing and validating transition readiness in individuals aged 12–26 years with a chronic illness between 2018 and 2022. Two reviewers independently selected articles for review and assessed quality of measurement properties.

**Results:**

22 studies met inclusion criteria reporting on 21 different tools. 9 studies reported on the development and evaluation of a new tool, and 13 reported on the adaptation, modification, and/or translation of an existing tool. Most adapted tools were translations and adaptations of the Transition Readiness Assessment Questionnaire (TRAQ) (n = 7). While some of these studies demonstrated sufficient internal consistency and structural validity, few met the COSMIN criteria for reliability and hypothesis testing and none met the criteria for cross-cultural validity. Criterion validity and measurement error were not assessed in any studies.

**Conclusion:**

Many new transition readiness measures continue to be developed in recent years, yet few have undergone rigorous psychometric evaluation. The TRAQ was the existing measure most often used as a model for developing new or modified tools. There remains a clear need for further validation of existing measures of patients’ readiness to transition as opposed to continuing to develop new measures.

## 1 Introduction and Background

The transition from pediatric to adult care settings for adolescents and young adults living with chronic conditions can be challenging and has been associated with declines in health and access to care.^1^, ^2^ Transition has been defined by the Society for Adolescent Medicine as the “purposeful, planned movement of adolescents and young adults with chronic physical and medical conditions from child-centred to adult-oriented health care systems”.^3^ Successful transition requires that youth acquire skills in self-care, health care decision-making, and self-advocacy that will prepare them to take more responsibility for their health and health care.^4^, ^5^ Greater involvement in self-management can facilitate successful transition to adult health care.^6^ Transition readiness captures the “process of building the capacity of adolescents and those who are involved in their medical care to prepare for, enter, continue and complete transition”.^7^ Readiness can be measured by assessing the adolescent’s desire and ability to develop autonomy and independently manage their health.^8^ Readiness assessments can be used in clinical settings to identify adolescents at risk for poor self-management, as well as promote increased self-management through follow-up education based on assessment results. Readiness assessments are also commonly applied to measure the effectiveness of transition preparation interventions for quality improvement or research purposes.

To facilitate the implementation of transition recommendations in clinical practice, the National Health Care Transition Center developed the Six Core Elements of Health Care Transition.^9^ The Six Core Elements are health care quality indicators that provide a structured approach to facilitate transition improvement. The third element, Transition Readiness, includes regular transition readiness assessments, beginning at age 14, to identify self-care needs and goals. The Six Core Elements have been shown to facilitate an effective transition process in subspecialty practices,^10^ a managed care plan,^11^ a children’s hospital,^12^ and a combined internal medicine and pediatrics residency program.‍^13^ In addition, a recent position statement by the Canadian Pediatric Society outlined the need to regularly assess transition readiness with both patients and caregivers in order to support a successful transition.^1^ Well-developed and validated measures of patients’ transition readiness are critical, both for use in clinical settings and to rigorously evaluate transition support programs for the purposes of research and health care quality improvement.

The need for readiness measurement as a key component of successful transitions has stimulated a rapid expansion of research aimed at developing and validating new (and existing) transition readiness measures. Although there are generic transition readiness measurement tools that have been developed and well-validated using a variety of validation metrics (i.e. the Transition Readiness Assessment Questionnaire (TRAQ),^14^ the Transition-Q,^15^ etc.), this field of research continues to grow. New tools are continually being created and evaluated, often with a focus on developing disease-specific measures and making tools available in languages other than English. Building on two previous foundational systematic reviews of transition readiness measures by Stinson^8^ (searched up to year 2014) and Parfeniuk^16^ (searched up to year 2018), the goal of this scoping review was to: (1) identify any newly developed and validated measures between 2018 and 2022 for the assessment of transition readiness for youth with chronic conditions; and (2) evaluate their measurement properties and identify gaps in measurement testing.

## 2 Methods

Electronic searches were conducted by a Library Information Specialist familiar with the field. The search date parameters were from April 1, 2018 (the day after the search dates in previous review^16^) until June 6 2022, and included the following databases Medline, CINAHL, Web of Science, PsycInfo. Search strategy terms (including subject headings and MeSH terms) were based off an established search from a previous review,^8^ which included terms related to childhood chronic illnesses (i.e. “arthritis”, “juvenile diabetes”, “cerebral palsy”, “chronic disease”, etc.), terms related to transitions in care (i.e. “patient transfer”, “transition to adult care”) and terms related to transition readiness measurement (i.e. “questionnaires”, “survey*”). The full search strategy can be found in the supplemental material (Appendix 1. Sample search strategy). Reference lists from all identified appropriate papers and review papers were examined and then hand searched for additional relevant studies. Finally, the review was designed and conducted in accordance with the Preferred Reporting Items for Systematic Reviews and Meta-Analyses (PRISMA) extension for Scoping Reviews (PRISMA-ScR)^17^.

### 2.1 Study Selection. To be included in the review, articles describing transition readiness measures had to meet the following criteria

1. Published in a peer-reviewed journal.
2. Report on a measure developed for or assessed in individuals with a chronic illness (defined as “any medical condition lasting more than one year that impairs function and/or requires ongoing medical care”), aged 12–26 years, before, during or following the transition from pediatric to adult care.
3. Report on the development and/or validation of the measure.
4. Provide sufficient measurement data to facilitate application of the COnsensus-based Standards for the selection of health Measurement INstruments (COSMIN) updated criteria for good measurement properties checklist.^18^

The following article types were excluded from the review:

1. Review articles, which were hand searched to find relevant studies.
2. Guidelines, dissertations, reports, commentaries, or abstracts.
3. Articles not published in English.

### 2.2 Review process

All search titles and abstracts were independently rated for relevance by at least two reviewers (TK, FN, LK) using Covidence for reference management and reviewing. Articles selected as relevant were compared between the two reviewers. Following discussion to resolve disagreement, consensus was reached on the articles selected for review. No attempt was made to locate unpublished material or contact researchers for unpublished studies or data (e.g., dissertations or conference proceeding abstracts). Relevant additional studies identified from references were reviewed.

### 2.3 Data charting and extraction

A standardized data extraction form was developed and tailored to the unique research question; this was utilized to assess the target population, measurement concepts, and measurement properties of each measure. At least two reviewers independently extracted information on each tool, including the name of the tool, the authors, whether it was a newly developed tool or a modification of an existing tool, target population, measurement concepts, number of scales/subscales/items, measurement properties evaluated (according to the COSMIN taxonomy^18^), and results. The extraction form was pilot tested prior to data extraction, and information extracted was compared by the reviewers to ensure level of agreement.

### 2.4 Measurement properties

Studies were reviewed using a standardized strategy to evaluate the measurement properties of existing measurements of transition readiness. The COnsensus-based Standards for the selection of health Measurement INstruments (COSMIN) updated criteria for good measurement properties was applied to rate the sufficiency of each measurement property^18^ (FN, LK). COSMIN taxonomy was also used to classify the various psychometric testing completed in each study (i.e. if a study reported completing “face validity” this was translated to “content validity” as per the COSMIN taxonomy).^18^ The COSMIN criteria were developed using Delphi methodology, and assesses the sufficiency of the nine properties of the instrument: internal consistency, reliability, measurement error, content validity, construct validity, criterion validity and responsiveness.^18^ The COSMIN criteria rate each result as sufficient (“+”), insufficient (“-”) or indeterminate (“?”) based on reported results^18^; each subscale is considered as its own scale and therefore each is evaluated independently. For the purposes of this paper, for a measurement property to be considered sufficient, all subscales had to receive a “+” rating. For this review, we based assessment of internal consistency on the reported Cronbach’s alpha (>0.7) in combination with demonstration of sufficient structural validity (i.e. only measures with sufficient structural validity can achieve a (“+”) rating for internal consistency). As there is no gold standard in transition measurement, all studies investigating criterion validity by correlating instruments and/or subscales were evaluated as construct validity.

The criteria for hypotheses testing for construct validity was based on the updated COSMIN criteria (see Appendix 2. COSMIN definitions of domains, measurement properties and aspects of measurement properties and Appendix 3. Criteria for good measurement properties). A correlation of at least 0.6 was hypothesized between different transition readiness measures and/or their subscales. Hypotheses for comparisons across time (responsiveness) were evaluated as effect sizes, of < 0.20 were expected for time points completed with 6 months, and ≥ 0.20 for time points beyond 6 months. Construct validity was deemed sufficient (“+”) if > 75 % of the results were in line with this hypothesis. If construct validity was assessed by comparing sex, age, or severity of disease it was not conceptualized as “construct validity” for the purpose of this manuscript; similarly, hypothesis testing for translations of measures were not reported (i.e. left blank) unless they were being correlated with another measure. For studies where the psychometric properties (reliability, hypothesis testing, cross-cultural validity, responsiveness) were not reported for each subscale identified during structural validity, we evaluated based on the total scale scores. Finally, content validity was not formally evaluated as many of the scales were in different languages, however a brief description of the content validity process (when reported) was included to highlight key elements of the tool development (i.e., discussion and validation with patients, experts, etc.).

## 3 Results

**Fig. 1:**
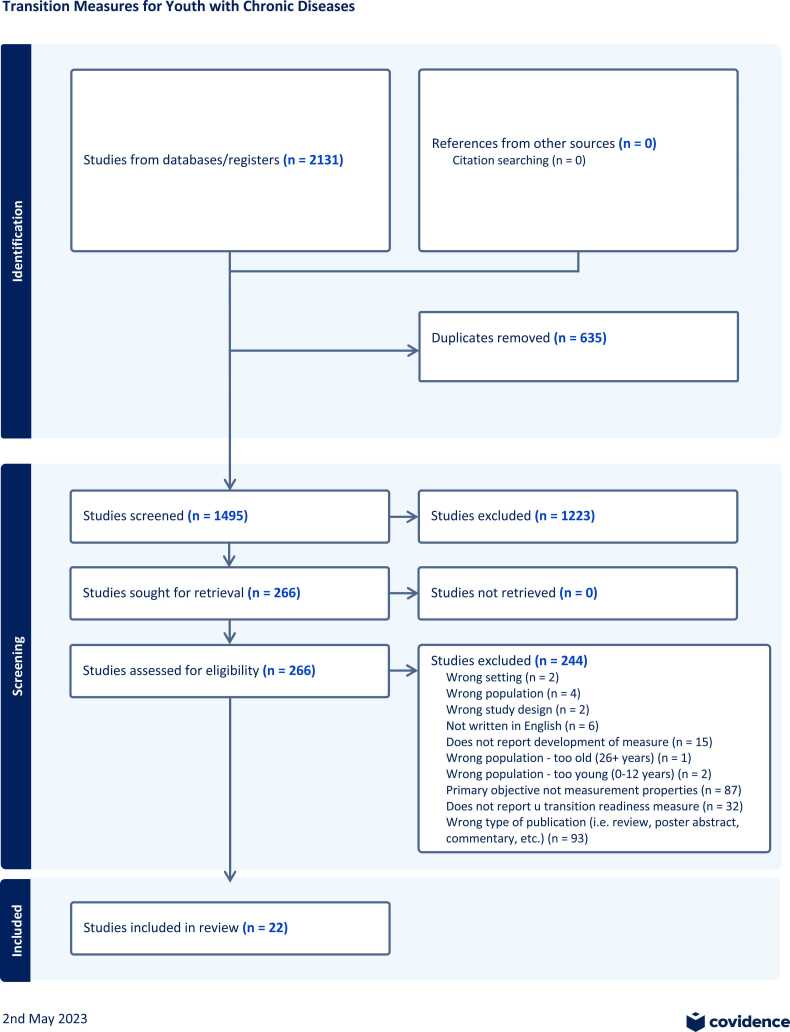
PRISMA flow diagram.

A total of 2131 abstracts were identified from the electronic searches (Fig. 1. PRISMA Flow Diagram). Duplicates accounted for 635 abstracts, leaving 1496 to be screened. Based on screening results, 179 full-text articles were assessed for eligibility. Of these, 157 articles were excluded and 22 articles reporting on 21 tools were included for extraction.

### 3.1 Study participants and design

An overview of all study characteristics can be found in Table 1. The majority of studies were conducted in the United States (n = 9)^19^, ^20^, ^21^, ^22^, ^23^, ^24^, ^25^, ^26^, ^27^, Canada (n = 2)^28^, ^29^ and Turkey (n = 2)^30^, ^31^, with the remaining studies conducted in Germany and Austria (n = 1)^32^, Italy (n = 1)^33^, Chile (n = 1)^34^, Norway (n = 1)^35^, China (n = 1)^36^, Japan (n = 1)^37^, South Africa (n = 1)^38^, Brazil (n = 1)^39^ and Taiwan (n = 1).^40^ Studies primarily recruited participants from hospital settings (n = 20), with only a small number focusing on community settings such as summer camps (n = 2). Patient populations included those experiencing a range of childhood onset chronic conditions (n = 6),^24^, ^29^, ^32^, ^34^, ^36^, ^37^ or focused on specific populations such as youth with type 1 diabetes (n = 6),^22^, ^23^, ^25^, ^27^, ^30^, ^31^, ^35^ spina bifida (n = 3),^19^, ^20^, ^26^ congenital heart disease (n = 2),^33^, ^40^ rheumatic disease (n = 2),^28^, ^39^ epilepsy (n = 1),^27^ HIV (n = 1)^38^ and sickle cell disease (n = 1).^21^ All studies were required to include a validation component to meet inclusion criteria. Most studies did not report specific use of a theory or conceptual model; however the most commonly cited model was the Transtheoretical Model (also known as the Stages of Change model)^41^ (n = 5). Sample sizes varied widely, especially depending on the phase of the study, and typically ranged from 76 to 500 participants for psychometric testing, with most studies having near equal gender split or slightly more female participants than male (n = 12).

**Table 1:** Overview of Studies of Transition Readiness Measures.

Author	Year	Transition Measure Name	Country	Setting	Population/ Disease Group	Study Design	Theory/ Conceptual Model	Total Sample Size	Age (mean ± sd /median/n ( %) and range)	% Female
Anelli et al.	2019	Brazilian-Portuguese TRAQ	Brazil	Hospital	Chronic rheumatic disorders	Development, Evaluation, Validation	Not specified	142	17.0 ± 2.2 14–21	75
Chen et al.	2017	HNS-CHD: The Healthcare Needs Scale for Youth with CHD	Taiwan	Hospital	Congenital heart defects	Evaluation, Validation	Not specified	500	18.8 ± 2.6 15–24	48
Clark et al.	2020	EpiTRAQ	United States	Hospital	Epilepsy	Development, Evaluation, Validation	Not specified	Initial validation: 302 Repeat validation: 381 Reliability: 153	Initial validation^a^: 302 Repeat validation^b^: 381 Reliability^c^: 153	Initial validation: 54 Repeat validation: 52 Reliability: 54
Culen et al.	2019	TRAQ-GV-15	Austria and Germany	Hospital	Chronic conditions (broadly)	Development, Evaluation, Validation	The TRAQ used the Transtheoretical Model	172	16.9 ± 1.8 14–23	60
Dellafiore et al.	2020	I-HNS-CHD-s: Italian-Short Version of Healthcare Needs Scale for Youth with CHD	Italy	Hospital	Congenital heart defects	Evaluation, Validation	Not specified	Sample A: 152 Sample B: 141	Sample A: 18.17 ± 2.12 Sample B: 17.50 ± 3.43	Sample A: 41.5 Sample B: 44.7
Funes et al.	2020	Not named	Chile	Hospital	Chronic conditions (broadly)	Evaluation, Validation	Stages of the transition theoretical model of change	168	14.4 ± 1.66 12–19	66
Goethals et al.	2020	RISQ-T: Readiness for Independent Self-Care Questionnaire Adolescent (RISQ-T)	United States	Hospital	Diabetes	Development, Evaluation, Validation	Not specified	178	14.9 ± 1.3 13–17	48
Hodnekvam et al.	2020	Not named	Norway	Community (national childhood registry)	Type 1 diabetes	Development, Evaluation, Validation	Not specified	321	22.9 ± 1.2	57
Johnson et al.	2019	TRAQ-SB	United States	Hospital	Spina bifida	Development, Evaluation, Validation	Stages of change model	90	12–17 years: 54.4 % 18–25 years: 45.6 %	50
Kızıler et al.	2019	Turkish version of Mind the Gap scale	Turkey	Hospital	Diabetes	Development, Evaluation, Validation	Multiple Inconsistency Theories	109	15.2 ± 1.44 14–21	46
Kızıler et al.	2019	TRAQ - Turkish version	Turkey	Hospital	Type 1 diabetes	Evaluation, Validation	Stages Of Change And Transtheoretical Model	109	15.2 ± 1.44	46
Loew et al.	2020	Self-Management Skills Checklist	United States	Hospital	Sickle Cell Disease	Development, Evaluation, Validation	Not specified	114	15.6 ± 1.03	41
Ma et al.	2020	STARx Questionnaire- Chinese	China	Hospital	Rheumatic diseases, Renal diseases and diabetes	Evaluation, Validation	Not specified	Sample 1 (8–11 year): 244 Sample 2 (12–18 years): 227 Total sample: 471	Sample 1: 9.61 ± 1.17 Sample 2: 13.3 ± 1.35 Total sample: 11.41 ± 2.25 8–18	Sample 1: 41 Sample 2: 41 Total sample: 41
Mellerio et al.	2019	French Good2Go	Canada and France	Hospital	type 1 diabetes, epilepsy, cystic fibrosis, juvenile idiopathic arthritis, or inflammatory bowel disease	Evaluation, Validation	Not specified	RMEF Study: 223 Pass’Age study: 98	RMEF Study: 16.0 ± 1.4 14–18 Pass’Age study: 17.3 ± 1.2 16–21	RMEF Study: 50 Pass’Age study: 46
Nazareth et al.	2018	STARx Questionnaire	United States	Hospital and Community (summer camp)	Chronic conditions (broadly)	Validation	Not specified	455	12.2 ± 2.53	57
Papadakis et al.	2021	Diabetes Skills Checklist	United States	Community (diabetes summer camp)	Type 1 diabetes	Development, Evaluation, Validation	Not specified	1155 adolescents	14.4 ± 1.51 12–18	54
Pierce et al.	2020	HCTOI: Healthcare Transition Outcomes Inventory	United States	Hospital	Type 1 diabetes	Evaluation, Validation	Not specified	128	22.2 ± 1.92 18–25	75
Sato et al.	2020	Japanese TRAQ	Japan	Hospital and Community (Member of Patient Association for Congenital Heart Disease)	Childhood onset Chronic conditions (broadly)	Development, Evaluation, Validation	Not specified	Phase 1: 6 Phase 2: 76	Phase 1 – Females: 18.2 years Male: 19 years Range: 18–20 Phase 2 – Females: 18.2 years Males: 17.8 years	Phase 1: 83 Phase 2: 47
Sawin et al.	2018	AMIS II: Adolescent/ Young Adult Self-Management and Independence Scale II	United States	Hospital	Spina bifida	Development, Evaluation, Validation, Pilot testing	(1) Ecological model of Adaptation in Spina Bifida, (2) Individual and Family Theory of Self-Management, and (3) International Classification of Functioning, Disability, and Health	Feasibility: 9 Validation: 201 Pilot: 61	Feasibility: 17.8 ± 4.7 12–25 Validation: 15.6 ± 3.25 12–25 Transition: 21.0 ± 2.1 21–25	Feasibility: 55 Validation: 52 Transition: 60
Spiegel et al.	2021	RACER: Readiness for Adult Care in Rheumatology	Canada	Hospital	Chronic rheumatic disorders	Development, Evaluation, Validation	Not specified	Content Validation: 30 Psychometric: 96	Content Validation: 14.5 Psychometric: 17.5 15–20	Content Validation: 73 Psychometric: 68
Wood et al.	2019	TRAQ-SB	United States	Hospital	Spina bifida	Validation	Stages of change model	90	12–17 years: 54.4 % 18–25 years: 45.6 %	50
Zanoni et al.	2021	HARTS: HIV adolescent readiness for transition scale	South Africa	Hospital	HIV	Development, Evaluation, Validation, Pilot testing	Not specified	Scale Development: 20 Psychometric Testing: 131 Pilot testing: 199	Psychometric Testing: 14 median age 13–15 Pilot testing: 13 median age 12–13	49

a Initial validation age breakdown by percentage of sample: 16–18 years – 31 %; 19–21 years –27.5 %; 22–26 years –41.4 %.

b Repeat validation age breakdown by percentage of sample: 16–18 years – 30.7 %; 19–21 years –27.0 %; 22–26 years –42.3 %.

c Reliability age breakdown by percentage of sample: 16–18 years – 28.8 %; 19–21 years –29.4 %; 22–26 years –41.8 %.

### 3.2 Characteristics of transition readiness measures

Of the 22 included articles, 9 were reporting on the development and evaluation of a new tool, and 13 were reporting on the adaptation, modification, and/or translation of an existing tool. Most adapted tools were translations and adaptations of the Transition Readiness Assessment Questionnaire^14^ (TRAQ) (n = 7), or adaptations of the Self-Management and Transition to Adult care with Treatment questionnaire^42^ (STARx) (n = 2), Mind the Gap^43^ (n = 1), Good2Go^44^ (n = 1), the Adolescent/Young Adult Self-management and Independence scale 1^45^ (AMIS 1) (n = 1) or the Healthcare Needs Scale for Youth with Congenital Heart Disease scale^40^ (HNS-CHD) (n = 1). All tools were reported by participant self-report. Three tools also had a parent-report component, although we did not include psychometric evaluations of parental-report measures in this review. The number of items in each questionnaire ranged from 11 to 98, and the number of sub-scales ranged from 2 to 7. Subscales were typically divided by common content area such as disease knowledge, self-management, autonomy or independence, and communication or self-advocacy. Only 5 studies discussed the process of content validation for the tool, and of these, only two studies included adolescents in the validation process.^28^, ^37^ An overview of transition readiness measure characteristics including a brief description of content validity (when applicable) can be found in Table 2.

**Table 2:** Characteristics of Measures.

Transition Measure Name	Type of Measure	Changes from Original	Tool Administration Method	Number of questions	Subscales	Psychometric properties	Content Validity Participants	Brief description of content validity process (if applicable)
Not named; Funes et al.	New tool	N/A	Participant self-report	24	6 subscales: Daily activities, Aspects of my illness, Management and use of medications, Practical aspects of health care, Involvement in the health checkup, and Transfer	Structural validity using principal component analysis Content validity Internal consistency	Healthcare providers	11 experts participated: 3 adult specialists (internists), 6 adolescent specialists (adolescent pediatricians), and 2 experts in children’s medicine (pediatricians). Experts independently reviewed each item and assessed their relevance to the transition process using a 4-point scale; were also asked to evaluate the instrument qualitatively on the different dimensions and items. Item content validity index was calculated.
Not named; Hodnekvam et al.	New tool	N/A	Participant self-report	98	7 subscales: Enough time, Understandable, Competence, Adult individualized care, Identified goal, Individualized advice, availability of support	Structural validity using exploratory factor analysis Construct validity Internal consistency Reliability		
Diabetes Skills Checklist	New tool	N/A	Participant self-report	14	Zero (no subscales)	Structural validity using exploratory factor analysis Construct validity Internal consistency		
HARTS: HIV adolescent readiness for transition scale	New tool	N/A	Participant self-report	16	4 subscales: Disclosure, Health navigation, Self-advocacy, Health Literacy	Structural validity using confirmatory factory analysis Internal consistency Reliability		
HCTOI: Healthcare Transition Outcomes Inventory	New tool	N/A	Participant self-report	34	5 subscales: Navigation, Self-management, Integration, Ownership, and Parental Support	Structural validity using confirmatory factory analysis Construct validity Internal consistency		
RACER: Readiness for Adult Care in Rheumatology	New tool	N/A	Participant self-report	32	6 subscales: General Knowledge, Knowledge About Medications, Planning For Adult Life, Managing Your Health Condition, Standing Up For Yourself, Knowing How to Get Around the Healthcare System	Construct validity Content validity Internal consistency Reliability Responsiveness	Healthcare providers, Researchers, Adolescents, Parents/Caregivers	Descriptive study design was used to determine comprehensiveness, relevance, and understanding of the RACER. Clinicians and researchers with experience in adolescent rheumatic medicine, as well as adolescents (aged 12–18 years) and one of their parents were invited to test the content validity of the instrument. Participants rated the relative importance of each domain and item; also were asked about the clarity of the content, meaning, wording, and intelligibility of items and whether they felt that there were any missing domains, and / or items. The importance of each domain and item to the process of health care transition was rated on a 5-point Likert scale and the content validity ratio (CVR) was computed.
RISQ-T: Readiness for Independent Self-Care Questionnaire Adolescent (RISQ-T)	New tool	N/A	Participant self-report	20	3 subscales: Knowledge, Behaviour and Perceived Importance	Internal consistency Construct validity Reliability		
HNS-CHD: The Healthcare Needs Scale for Youth with CHD	New tool	N/A	Participant self-report	35	3 subscales: Health management, Healthy policy, Individual and interpersonal relationships	Structural validity using exploratory factor analysis Construct validity Internal consistency		
I-HNS-CHD-s: Italian-Short Version of Healthcare Needs Scale for Youth with CHD	Translation and adaptation of HNS-CHD	Reduction to 14 items original has 35 items; Modified to 4 subscales versus 3 subscales in original	Participant self-report	14	4 subscales: Health-care education, clinical support, emotional support, continuum of care	Structural validity using exploratory factor analysis Content Validity Internal consistency	Healthcare providers	Panelists were 16 experts (nurses and physicians specialized in CHD transition care and research methodology) rated the pertinence and the relevance of each item with the objective of its measurement. Content Validity Ratio (CVR) was used to assess the pertinence through a three-point ordinal scale and Content Validity Index (CVI) to assess relevance. Qualitative content validity (i.e., face validity) was determined based on the same expert panelists’ understanding of the items and their views about the overall concept that they purported to measure. Questions explored clarity of wording used for each item and identified areas of ambiguity. Answers were analyzed using a narrative approach to summarize.
AMIS II: Adolescent/ Young Adult Self-Management and Independence Scale II	Adapted version of the AMIS I	Modified to 17 items, AMIS I has 10 items	Structural interview completed by participant and rated by healthcare provider	17	2 subscales: Independent living and self-management, Condition self-management	Structural validity using exploratory factor analysis and confirmatory factory analysis Construct validity Internal consistency Reliability		
French Good2Go	Translation and adaptation of Good2Go	Translation to French; one item was removed	Participant self-report	21	3 subscales: Health self-advocacy, Knowledge about chronic conditions, Self-management skills	Structural validity using exploratory factory analysis Internal consistency Reliability		
Mind the Gap - Turkish version	Translation of the Mind the Gap to Turkish	No changes beyond translation	Participant self-report	22	2 subscales: Mind the Gap scale 1 and Mind the Gap scale 2	Structural validity using exploratory factory analysis Content Validity Internal consistency Reliability Construct validity		
STARx Questionnaire; Nazareth et al.	Existing tool was modified and re-evaluated in a new sample	Reduction to 13 items original had 18 items; Modified to 3 subscales while the original has 6	Participant self-report	13	3 subscales: Disease Knowledge, Self-management and Provider Communication	Structural validity using principal component factory analysis Internal consistency Construct validity		
STARx Questionnaire-Chinese	Translation and adaptation of STARx Questionnaire	One question was reworded to suit the Chinese context; Modified to 4 subscales while the original has 6	Participant self-report	18	4 subscales: Medication management, Healthcare engagement, Provider communication, Disease knowledge	Structural validity using exploratory factor analysis and confirmatory factor analysis Content validity Internal consistency	Unspecified	The item and scale-level content validity average scores (S-CVI/Ave) obtained from the expert review of the STARx-C Questionnaire were calculated. In the STARx-C Questionnaire, all items that were considered very important and meaningful were retained. Only one item ‘How often did you make your own appointments?’ was revised to ‘How often did you make appointments online or register in the outpatient programme by your own?’ according to the health care system in China. The overall S-CVI/Ave of the expert content validity scores of the STARx-C Questionnaire was 0.96.
Self-Management Skills Checklist	New tool (Development guided by items in TRAQ)	N/A	Participant self-report	22	2 subscales: Adolescent skills and Adolescent knowledge	Structural validity using exploratory factor analysis Content Validity Internal consistency Responsiveness	Healthcare providers	Transition program team members (psychologists, psychology fellow, hematologist, nurse case managers, mid-level provider, social worker, and licensed teacher) revised items to coincide with the SCD transition program education curriculum (eg, medical history knowledge, pain management, and disease-specific knowledge). After the initial list of items were created the transition program team members reviewed and revised the items again for face validity and readability.
TRAQ-GV-15	Translation and adaptation of TRAQ	Reduction to 15 items, original TRAQ has 20 items; Modified to 3 subscales while the original has 5	Participant self-report	15	3 subscales: Autonomy, Health Literacy and Adherence	Structural validity using exploratory factor analysis Internal consistency		
TRAQ-SB (Johnson et al., 2019)	Translation and adaptation of TRAQ	Reduction to 11 items original TRAQ has 20 items	Participant self-report	11	5 subscales: Managing Medication, Appointment Keeping, Tracking Health Issues, Talking With Providers and Man-aging Daily Activities	Structural validity using principal component factor analysis Construct validity Internal consistency		
TRAQ-SB (Wood et al., 2019)	Translation and adaptation of TRAQ	Reduction to 11 items original TRAQ has 20 items	Participant self-report	11	5 subscales: Managing Medication, Appointment Keeping, Tracking Health Issues, Talking With Providers and Man-aging Daily Activities	Predictive validity		
Brazilian-Portuguese TRAQ	Translation and adaptation of TRAQ	One question was reworded to suit the Brazilian context	Participant self-report	20	5 subscales: Managing Medication, Appointment Keeping, Tracking Health Issues, Talking With Providers and Man-aging Daily Activities	Structural validity using confirmatory factory analysis Internal consistency		
EpiTRAQ	Translation and adaptation of TRAQ	Modified to include 15 items specific to epilepsy based on American Academy of Neurology guidelines	Participant self-report	35	5 subscales: Managing Medication, Appointment Keeping, Tracking Health Issues, Talking With Providers and Man-aging Daily Activities	Internal consistency Responsiveness		
Japanese TRAQ	Translation and adaptation of TRAQ	Three questions were reworded to suit the Japanese context; Modified to 4 subscales while the original has 5	Participant self-report	23	4 subscales: Managing Medications, Tracking Health Issues, Appointment Keeping, Talking with Providers	Content validity Internal consistency	Adolescents	Preliminary survey was conducted to confirm the face validity. Participants were recruited via snowball sampling: (i) aged 16–20 years at the time of the survey, (ii) having a childhood-onset chronic illness presumed to require continued treatment and follow-up into adulthood or longer, (iii) capable of responding to the questionnaire by themselves (or with assistance in writing), and (iv) provided consent to participate in the study. Participants filled out the face sheet and the Japanese TRAQ (draft) in a self-administered fashion, either face-to-face or online. The face sheet contained questions regarding participant background (e.g. sex, age, disease group, and disease name). After that, we asked participants if anything was unclear and how they understood the meaning of each question. We also measured the time required to respond to the questionnaire and the presence or absence of missing data. Based on this preliminary survey, we verified the face validity of the Japanese TRAQ (draft) and used it as the final version in the main survey.
TRAQ - Turkish version	Translation of the TRAQ to Turkish	No changes beyond translation	Participant self-report	20	5 subscales: Managing Medication, Appointment Keeping, Tracking Health Issues, Talking With Providers and Man-aging Daily Activities	Structural validity using exploratory factory analysis Content validity Construct validity Internal consistency Reliability	Healthcare providers	The translation and back-translation method was used with expert opinions to determine content validity. First, three English language experts and two native Turkish researchers who speak English fluently independently translated the original scale into Turkish. The translations were combined and prepared as a single text that was then retranslated into English by two other English-language experts. Next, the expert opinions of two nursing academicians experienced in care transition and research methods, a biostatistician, and a pediatric endocrinologist were incorporated. The scale was finalized; no more changes were made, and all 20 items in the questionnaire were included in the final form of the Turkish TRAQ.

### 3.3 Measurement properties of transition readiness measures

An overview of the evaluation of measurement properties of each measure using the COSMIN criteria can be found in Table 3, with additional information on the results for each measurement property in Supplementary Table 1 (Results of studies to determine COSMIN Criteria for Good Measurement). Most studies conducted a range of psychometric testing on the transition readiness measures, which was primarily composed of validity and internal consistency, with some studies including reliability and three including responsiveness. All studies reported on internal consistency (n = 21) but only 6 measures (AMIS II, Diabetes Skills Checklist, HNS-CHD, STARx-Chinese, TRAQ-SB, and the Turkish version of the Mind the Gap scale) demonstrated sufficient internal consistency according to the COSMIN Criteria for Good Measurement Properties. Most studies reported on structural validity (n = 17), however only 10 measures demonstrated sufficient structural validity (AMIS II, Brazilian-Portuguese TRAQ, Diabetes Skills Checklist, HCTOI: Healthcare Transition Outcomes Inventory, HNS-CHD, I-HNS-CHD-s, STARx-Chinese, TRAQ - Turkish version, TRAQ-SB, and the Turkish version of Mind the Gap scale). 10 studies reported on reliability, with 5 meeting the COSMIN criteria for sufficiency (AMIS II, French Good2Go, RACER, TRAQ - Turkish version, and the Turkish version of the Mind the Gap scale), and 11 studies reported on hypotheses testing for construct validity with only two meeting the COSMIN criteria for sufficiency (RACER, TRAQ-SB). Only three studies reported on responsiveness, with only one measure evaluated as sufficient (RACER). As noted in Table 3, no studies reported on cross-cultural validity, criterion validity (as there is no gold standard comparator) or measurement error. Overall, the AMIS-II, the TRAQ-SB and the RACER were the strongest tools, demonstrating sufficiency in 3/5 of the reported COSMIN criteria, and the Diabetes Skills Checklist, HNS-CHD, TRAQ - Turkish version, Turkish version of Mind the Gap scale and the STARx-Chinese demonstrated sufficiency in 2/5 of the reported COSMIN criteria.

**Table 3:** Summary of COSMIN Criteria for Good Measurement.*

	Transition Measure Name	Structural Validity	Internal Consistency	Reliability	Hypotheses testing for construct validity^†^	Responsiveness
1	AMIS II: Adolescent/ Young Adult Self-Management and Independence Scale II	Two factor structure: +	Factor 1 Independent Living: + Factor 2 Condition Self-Management: +	Total Scale: +	a: 1 + /4–	
2	Brazilian-Portuguese TRAQ	+	Total scale: + Factor 1 Managing Medications: – Factor 2 Managing Daily Activities: – Factor 3 Tracking Health Issues: – Factor 4 Appointment Keeping: –			
3	Diabetes Skills Checklist	One factor: +	Total Scale: +		a: 1 + /1–	
4	EpiTRAQ		?			?
5	French Good2Go	Three factors: –	Domain 1: – Domain 2: – Domain 3: –	Domain 1: + Domain 2: + Domain 3: +		
6	HARTS: HIV adolescent readiness for transition scale	Four factors: –	Total scale: –	?		
7	HCTOI: Healthcare Transition Outcomes Inventory	Five factors: +	Factor 1 Continuity of care: – Factor 2 Collaborative relationships: + Factor 3 Integration: + Factor 4 Ownership: + Factor 5 Parental support: +		a: 2 + /8– b: 4 + /6–	
8	HNS-CHD: The Healthcare Needs Scale for Youth with CHD	Three factors: +	Factor 1 Health management: + Factor 2 Health policy: + Factor 3 Individual and Interpersonal Relationships: +		a: 1–	
9	I-HNS-CHD-s: Italian-Short Version of Healthcare Needs Scale for Youth with CHD	Four factors: +	Total Scale: + Factor 1 Healthcare education: + Factor 2: Clinical support: + Factor 3 Emotional Support: – Factor 4 Continuum of care: +			
10	Japanese TRAQ		?			
11	Not named; Funes et al.	Two factors: –	Factor 1: – Factor 2: –			
12	Not named; Hodnekva et al.	?	?	Factor 1 Paediatric care doctor: + Factor 2 Paediatric care nurse: + Factor 3 Pediatric individualized care: + Factor 4 Prepare for transition: + Factor 5 Adult care doctor: + Factor 6 Adult care nurse: + Factor 7 Adult individualized care: –	b: 4 + /5–	
13	RACER: Readiness for Adult Care in Rheumatology		?	Total scale: +	+	+
14	RISQ-T: Readiness for Independent Self-Care Questionnaire Adolescent		?	Total scale: –	–	
15	Self-Management Skills Checklist	?	?			–
16	STARx Questionnaire; Nazaretha et al.	?	?		c: 2 + /1–	
17	STARx Questionnaire-Chinese	Four factors: +	Factor 1 Medicaiton Management: + Factor 2 Healthcare Engagement: + Factor 3 Provider Communication: + Factor 4 Disease Knowledge: +			
18	TRAQ - Turkish version	Five factors: +	Factor 1 Keeping Appointments: + Factor 2 Managing Medication: + Factor 3 Tracking Health issues: + Factor 4 Managing daily activities: + Factor 5 Talking with providers: +	Total scale: +	a: 1–	
19	TRAQ-GV-15	Three factors: –	Factor 1: – Factor 2: – Factor 3: –			
20	TRAQ-SB; Johnson et al., 2019 & Wood et al., 2019	One factor: +	Total scale: +		a: 6 +	
21	Turkish version of Mind the Gap^‡^	Best Care scale Three factors: +	Best Care scale Factor 1 Management of Environment: + Factor 2 Staff Characteristics: + Factor 3 Process Issues: +	Total questionnaire: +	Total questionnaire: +	
		Current Care scale Three factors: +	Current Care scale Factor 1 Management of Environment: + Factor 2 Staff Characteristics: + Factor 3 Process Issues: +			

† a: comparison with other instruments, b: comparison between subgroups, c: comparison to parent version of instrument.

‡: This questionnaire was broken into two scales, the structural validity and internal consistency were analyzed independently for each scale, as such results are reported separately.

* None of the studies reported on cross-cultural validity, criterion validity or measurement error so these items were removed from the table.

## 4 Discussion

This updated scoping review sought to build on existing literature reviews by identifying newly developed and validated measures for the assessment of transition readiness for youth with chronic conditions and appraise their measurement properties order to assess the degree to which these measures have been validated. We identified 21 transition readiness measures in 22 peer-reviewed articles. Similar to previous reviews,^8^, ^16^ none of the studies of transition readiness measures evaluated in this review consistently met standards for sufficient measurement properties. While some of these studies demonstrated sufficient internal consistency and structural validity, few provided evidence of hypothesis testing and cross-cultural validity. Furthermore, none of the studies assessed measurement error.

When discussing the importance of transition readiness measurement, it is necessary to define what characterizes a “good” transition. The Health Care Transition Research Consortium (HCTRC) recently sought to define successful health care transition outcomes utilizing a Delphi process with an interdisciplinary and international group of participants (parents, young adults, clinicians and researchers).^46^ Candidate outcomes were developed based on initial literature search and expert interviews, then further refined using two waves of a web-based survey. The 10 final outcomes were grouped into individual outcomes (quality of life, understanding health care condition and its complications, knowledge of medication, self-management, medication adherence, and understanding health insurance), health services outcomes (attending medical appointments, having a medical home, avoiding unnecessary hospitalizations) and a social outcome (having a social network). Similarly, Bailey et al. recently identified 169 quality indicators for transition to adult care for youth with chronic conditions.^2^ The most common measurement themes were transition education, continuity of care, satisfaction and self-efficacy, as well as indicators such as medication adherence and quality of life. While many of the transition measures in this scoping review also address many of these domains, there was a lack of patient and caregiver involvement in the development of these measures, which was also found in the review by Bailey et al.^2^ Going forward, these quality indicators and transition outcomes identified should be used to inform the development and testing of future transition measures, with an emphasis on including youth and caregiver perspectives.

In addition to this, Prior and colleagues have proposed leveraging the Triple Aim as an alternate framework for measuring transition outcomes^47^. Developed by the Institute for Health Care Improvement, the Triple Aim is organized around three goals: 1) improve the individual experience of health care, 2) improve the health of populations, and 3) reduce the per capita costs of care.^48^ Framing transition measurement in terms of the Triple Aim aligns transition improvement with broader initiatives to improve health care quality.^47^ However, in a systematic review of transition interventions, the authors found that there was little consistency in the reporting of transition outcomes, and that most studies examined only one domain of the Triple Aim using a variety of measures.^49^ While these assessments included some validated measures of self-care skills, none utilized validated measures of transition care experiences. Again, a focus on the most important transition outcomes is critical to advancing the field. When combined with measures of population health and health care costs, these studies have the potential to create strong evidence in support of structured transition support for youth with chronic conditions.

There is evidence that the use of transition readiness measurement tools can improve transitions and the provision of transitional care; while transition readiness assessments have not been robustly correlated with successful transfer to adult care, there is data to suggest that transition preparation programs correlate with improved care continuity and health outcomes. Cole et al. demonstrated that compared to patients receiving usual care, patients participating in a formalized transition clinic for patients with inflammatory bowel disease had decreased need for surgery, decreased hospital admissions, improved clinic attendance, better medication adherence, and increased likelihood of achieving maximum estimated growth potential at the end of adolescence.^50^ Interestingly, there was also a trend towards higher dependence on opiates and smoking among patients who did not receive transition support. However, the lack of correlation of transition readiness assessments with health and access-to-care outcomes suggests that existing transition readiness are not fully capturing factors critical to an effective transition. Future work in the development and validation of transition readiness measures should incorporate lessons learned from successful transition preparation programs, to ensure that factors which may be missing from current measures are addressed. These findings highlight the need for further research to develop comprehensive, psychometrically sound measures that can adequately predict transition success.

Overall, the sheer number of new and modified tools developed over this four-year period highlights the rapidly evolving nature of transition-related research, and specifically underscores the growing need for tailored, disease-specific transition readiness measurements across various disease groups. In addition to this, the broad range in country of origin and the strong focus on translation of existing readiness measures to new languages demonstrates the rising importance of improving transitions globally for youth with chronic conditions. Considering the quantity of existing transition readiness measures, the results of this review in combination with previous reviews suggest that it may be worthwhile for researchers to focus on continuing to establish validity of existing measures (when suitable to the population) as well as continuing to develop new measures with a specific emphasis on including youth and caregiver perspectives in the development process.

### 4.1 Limitations

There are several limitations of this review. First, only manuscripts available in English were included which limited the results. The literature search also included only articles published in peer-reviewed journals; therefore, measures nearing completion or awaiting publication were not included. This review also did not include measures related to transfer experience or satisfaction and excluded evaluations of parental reports of transition readiness. Finally, due to the nature and goals of a scoping review to provide an overview of the existing evidence, conducting assessments methodological limitations or risk of bias of studies was outside of the scope of this work.^51^ However, future research may seek to conduct these evaluations to compare new measures with existing measures of transition readiness. Future research may also wish to focus specifically on reviewing the development and validation of parent or caregiver-reported measures for transition readiness, as there is growing literature that supports the importance of including caregiver perspectives in transition preparation to support the transition process.^52^, ^53^, ^54^

## 5 Conclusion

In conclusion, adolescents and young adults with chronic conditions must inevitably transition from pediatric to adult-oriented health systems. This time period has been associated with declines in health among patients with a wide range of chronic conditions, often due to gaps in care or poor adherence.^55^, ^56^, ^57^, ^58^ Assessment of transition readiness in order to identify patients at risk for poor transition has been recommended in multiple policy statements and has been implemented as a part of multiple transition improvement interventions. While many transition readiness assessments have been developed and the number of new and adapted tools developed in the past 4 years has demonstrated the rapid proliferation of research in this field, there remains a clear need for further validation of existing measures of patients’ readiness to transition. These validated measures are crucial to prepare patients for adult-oriented care in the clinical setting, and to rigorously assess transition improvement programs and transition-related outcomes in research and health care quality-improvement settings. Recent research defining successful transition outcomes and identifying quality indicators should be leveraged in future development of transition readiness measures, with a strong emphasis on including youth and caregiver perspectives in the development of these measures.

## Funding source

No funding was secured for this study.

## CRediT authorship contribution statement

**Tieghan Killackey:** Conceptualization, Data curation, Formal analysis, Investigation, Methodology, Project administration, Resources, Software, Supervision, Validation, Visualization, Writing – original draft, Writing – review & editing. **Fareha Nishat:** Data curation, Formal analysis, Investigation, Methodology, Software, Validation, Visualization, Writing – review & editing. **Ellen Elsman:** Formal analysis, Investigation, Methodology, Software, Validation, Visualization, Writing – review & editing. **Erica Lawson:** Conceptualization, Formal analysis, Writing – original draft, Writing – review & editing. **Lauren Kelenc:** Formal analysis, Investigation, Methodology, Software, Validation, Visualization, Writing – original draft, Writing – review & editing. **Jennifer N. Stinson:** Conceptualization, Methodology, Project administration, Resources, Software, Supervision, Writing – original draft, Writing – review & editing.

## Declaration of Competing Interest

The authors declare that they have no known competing financial interests or personal relationships that could have appeared to influence the work reported in this paper.

## Figures and Tables

**Table:** 

#	Searches	Results
1	Arthritis, Juvenile Rheumatoid/	11269
2	exp Asthma/	137867
3	Cystic Fibrosis/	38567
4	exp Diabetes Mellitus/	480928
5	exp Epilepsy/	121671
6	exp Anemia, Sickle Cell/	24575
7	Cerebral Palsy/	22718
8	exp Spinal Dysraphism/	8756
9	exp Heart Defects, Congenital/	164393
10	Chronic Disease/	275849
11	(juvenile adj2 idiopath* adj2 arthritis).mp.	6479
12	asthma.mp.	189994
13	diabetes.mp.	705863
14	epilepsy.mp.	155028
15	(sickle adj2 cell).mp.	31402
16	(cerebral adj2 palsy).mp.	30401
17	(spina adj2 bifida).mp.	9361
18	(congenital adj2 heart adj2 disease*).mp.	35410
19	(cystic adj2 fibrosis).mp.	55551
20	(chronic* adj2 ill*).mp.	31600
21	or/1–20	1659483
22	Patient Transfer/	9432
23	Transition to Adult Care/	1937
24	transition*.mp.	506506
25	"Continuity of Patient Care"/	20371
26	(handoff* or handover*).mp.	3790
27	(patient* adj2 transfer*).mp.	19175
28	or/22–27	543968
29	exp Questionnaires/	1171551
30	exp Data Collection/	2455696
31	exp "Weights and Measures"/	248551
32	questionnaire*.mp.	878308
33	survey*.mp.	1174911
34	or/29–33	3314332
35	21 and 28 and 34	3836
36	(adolescen* or teen*).mp.	2265095
37	(young adj2 adult*).mp.	1067236
38	36 or 37	2770065
39	35 and 38	1515
40	limit 35 to ("adolescent {13–18 years)" or "young adult (19–24 years)")	1421
41	39 or 40	1515
42	limit 41 to ed=20180401–20220606	506

**Table:** 

Domain	Term		Definition
	Measurement property	Measurement property aspect	
Reliability			The degree to which the measurement is free from measurement error
Reliability (extended definition)			The extent to which scores for patients who have not changed are the same for repeated measurement under several conditions: e.g., using different sets of items from the same OMI (internal consistency); over time (test-retest); by different persons on the same occasion (inter-rater); or by the same persons on different occasions (intra-rater)
	Internal consistency		The degree of interrelatedness among the items
	Reliability		The proportion of the total variance in the measurements which is due to ‘true’^†^ differences between patients
	Measurement error		The systematic and random error of a patient’s score that is not attributed to true changes in the construct to be measured
Validity			The degree to which an OMI measures the construct(s) it purports to measure
	Content validity		The degree to which the content of an OMI is an adequate reflection of the construct to be measured
		Face validity	The degree to which (the items of) an OMI indeed seems to be an adequate reflection of the construct to be measured
	Construct validity		The degree to which the scores of an OMI are consistent with hypotheses (e.g., with regard to internal relationships, relationships to scores of other OMIs, or differences between relevant groups) based on the assumption that the OMI validly measures the construct to be measured
		Structural validity	The degree to which the scores of an OMI are an adequate reflection of the dimensionality of the construct to be measured
		Hypotheses testing	Idem construct validity
		Cross-cultural validity	The degree to which the performance of the items on a translated or culturally adapted OMI are an adequate reflection of the performance of the items of the original version of the OMI
	Criterion validity		The degree to which the scores of an OMI are an adequate reflection of a gold standard
Responsiveness			The ability of an OMI to detect change over time in the construct to be measured
	Responsiveness		Idem responsiveness
Interpretability*			The degree to which one can assign qualitative meaning (i.e., clinical or commonly understood connotations) to an OMI’s quantitative scores or change in scores

**Table:** 

Measurement property	Rating	Criteria
Structural validity*	+	**CTT:** EFA/PCA: factor loadings of each item on its factor is at least 0.30 *AND* maximum 10 % of the items load on more than one factor *AND* minimum explained variance is 50 % and structure is in line with the theory about the construct to be measured *OR* results on scree plot or Kaiser criterion (Eigenvalues >1) are in line with the theory about the construct to be measured CFA: CFI or TLI or comparable measure > 0.95 *OR* RMSEA < 0.06 *OR* SRMR < 0.08 **IRT/Rasch:** no violation of unidimensionality: CFI or TLI or comparable measure > 0.95 *OR* RMSEA < 0.06 *OR* SRMR < 0.08 *AND*no violation of local independence: residual correlations among the items after controlling for dominant factor < 0.20 *OR* Q3′s < 0.37 *AND*no violation of monotonicity: adequate looking graphs *OR* item scalability > 0.30 *AND*adequate model fit:IRT: χ^2^ > 0.01 Rasch: infit and outfit mean squares ≥ 0.5 and ≤ 1.5 *OR* Z-standardized values > −2 and < 2
	?	**CTT:** not all information for ‘+ ’ reported**IRT/Rasch:** model fit not reported
	-	Criteria for ‘+ ’ not met
Internal consistency	+	At least low evidence for sufficient structural validity *AND* Cronbach’s alpha(s) ≥ 0.70 for each unidimensional scale or subscale
	?	Criteria for “at least low evidence for sufficient structural validity” not met
	-	At least low evidence for sufficient structural validity *AND* Cronbach’s alpha(s) < 0.70 for each unidimensional scale or subscale
Reliability	+	ICC or (weighted) kappa or Pearson/Spearman correlation ≥ 0.70
	?	ICC or (weighted) kappa or Pearson/Spearman correlation not reported
	-	ICC or (weighted) kappa or Pearson/Spearman correlation < 0.70
Measurement error	+	SDC or LoA <MIC
	?	MIC not defined
	-	SDC or LoA > MIC
Hypotheses testing for construct validity	+	≥ 75 % of the results is in accordance with predefined hypotheses
	?	No hypotheses defined (by the review team)
	-	≥ 75 % of the results is not in accordance with predefined hypotheses
Cross-cultural validity\ measurement invariance	+	No important differences found between group factors (such as age, gender, language) in multiple group factor analysis *OR* no important DIF for group factors (McFadden’s R^2^ <0.02)
	?	No multiple group factor analysis *OR* DIF analysis performed
	-	Important differences between group factors *OR* DIF was found
Criterion validity	+	Correlation with gold standard ≥ 0.70 *OR* AUC ≥ 0.70
	?	Not all information for ‘+’ reported
	-	Correlation with gold standard < 0.70 *OR* AUC < 0.70
Responsiveness	+	≥ 75 % of the results is in accordance with predefined hypotheses *OR* AUC ≥ 0.70
	?	No hypotheses defined (by the review team)
	-	≥ 75 % of the results is not in accordance with predefined hypotheses *OR* AUC < 0.70

AUC = area under the curve, CFA = confirmatory factor analysis, CFI = comparative fit index, CTT = classical test theory, DIF = differential item functioning, EFA = exploratory factor analysis, ICC = intraclass correlation coefficient, IRT = item response theory, LoA = limits of agreement, MIC = minimal important change, PCA = principal component analyses, RMSEA: Root Mean Square Error of Approximation, SEM = Standard Error of Measurement, SDC = smallest detectable change, SRMR: Standardized Root Mean Residuals, TLI = Tucker‐Lewis index

*Standard 1 in Box 3 in the COSMIN Risk of Bias checklist^32^ was rated very good if CFA was performed, adequate if EFA was performed, doubtful if PCA was performed and inadequate if none of the previous was performed.

## Data Availability

No data was used for the research described in the article.

## Appendix 1 Sample search strategy

Ovid MEDLINE(R) and Epub Ahead of Print, In-Process, In-Data-Review & Other Non-Indexed Citations and Daily < 1946 to May June 03, 2022>.

## Appendix 2 COSMIN definitions of domains, measurement properties and aspects of measurement properties^12^

COSMIN: COnsensus-based Standards for the selection of health Measurement INstruments * Not considered a measurement property, but an important characteristic of a measurement instrument † The word “true” must be seen in the context of the CTT, which states that any observation is composed of two components—a true score and error associated with the observation. “True” is the average score that would be obtained if the scale were given an infinite number of times. It refers only to the consistency of the score and not to its accuracy.[70].
