# Supplementary material for: Transition readiness measures for adolescents with chronic illness: A scoping review of new measures
Source: Health Care Transit. 2023 Oct 12;1:100022. doi: 10.1016/j.hctj.2023.100022 (PMC11657346; doi:10.1016/j.hctj.2023.100022)
Supplement: Supplementary file 1 — Supplementary material [file mmc1.docx]

**Supplementary Table 1. Results of studies to determine COSMIN Criteria for Good Measurement**

| **Transition Measure Name** | **Structural Validity** | **Internal Consistency** | **Reliability** | **Hypotheses testing for construct validity** | **Cross-cultural validity/measurement invariance** | **Responsiveness** |
| --- | --- | --- | --- | --- | --- | --- |
| AMIS II: Adolescent/ Young Adult Self-Management and Independence Scale II | Two factor structure: CFI = 0.95; RMSEA = 0.057; χ2 = 180.41, p = 0.001; χ2/df = 1.7 | Subscale Cronbach's alpha:  Factor 1 (Independent Living Self-Management): 0.88  Factor 2 (Condition Self-Management): 0.80 | AYA total scale was 0.84, 95% CI (0.71, 0.92) | Pearson Correlation statistic for total scales  Adolescent Decision-Making Inventory: 0.42  Decision Making Subscale of the Adolescent Activities Inventory: 0.61  Chores subscale of the Adolescent Activities Inventory Parent report: 0.34  Self-care scale of the WeeFIMÒ Parent report: 0.29  Self-care scale of the WeeFIMÒ Adolescent report: 0.42 |  |  |
| Brazilian-Portuguese TRAQ | Two models assessed for confirmatory analysis, authors did not reveal information about models or the number of factors.  Model 1: χ2/df = 1.21; CFI = 0.89; TLI = 0.87; RMSEA = 0.04  Model 2: χ2/df = 1.03; CFI = 0.98; TLI = 0.98; RMSEA = 0.02 | Cronbach’s alpha for total scale: 0.776  Subscale Cronbach’s alpha:  Managing Medications = 0.56;  Managing Daily Activities = 0.59;  Tracking Health Issues = 0.60;  Appointment Keeping = 0.67 |  |  |  |  |
| Diabetes Skills Checklist | KMO = 0.95; χ2 = 19174.83 p < 0.001; one factor 14-item scale accounting for 56.72% of the variance; items factor loading ranging from 0.693-0.887 | -Cronbach’s alpha= (0.96) |  | Pearson Correlation statistic for total scale  Diabetes Skills Checklist – Parent: 0.87  Diabetes Strengths and Resilience Measure of Adolescents: 0.57 |  |  |
| EpiTRAQ |  | -Range of Cronbach's alpha values for both initial and repeat validation is (0.78, 0.95) and (0.77, 0.94), respectively |  |  |  | -Over 80% of adolescent and young adult patients gave themselves higher ratings on at least 3 items at Time 2, which occurred at least 6 months later than Time 1, and younger age was associated with an increase in scores from Time 1 to Time 2s. |
| French Good2Go | 3 factors (eigenvalue > 1) explaining more than 90% of item variance; loading were ≥ 0.44 on domain 1; ≥ 0.24 on domain 2; and ≥ 0.16 on domain 3 | Cronbach’s α  Domains 1: 0.85, Domain 2: 0.72, and Domain 3: 0.77 | Test-retest reliability for Domain 1: 0.76, Domain 2: 0.70, and Domain 3: 0.80 |  |  |  |
| HARTS: HIV adolescent readiness for transition scale | CFI = 0.93; RMSEA = 0.0885; loading were ≥ 0.347 on domain 1; ≥ 0.391 on domain 2; ≥ 0.315 on domain 3; ≥ 0.33 on domain 4 | Authors did not report internal consistency for each domain.  Cronbach’s alpha for the total final 15 question HARTS questionnaire was 0.78 | The mean score was 36.3 (standard deviation (SD) 7.3) on the first test  -36.9 (SD 6.7) on the second test (3 hours later)  -No statistical difference in the means (p=0.69) |  |  |  |
| HCTOI: Healthcare Transition Outcomes Inventory | Total scale (5 factors): CFI = 0.80, TLI = 0.78, RMSEA = 0.08, SRMR = 0.08  Continuity subscale: CFI = 0.94, TLI = 0.86, RMSEA = 0.08, SRMR = 0.06  Collaborative Relationship: CFI = 0.99, TLI = 0.96, RMSEA = 0.05, SRMR = 0.04)  Integration: CFI = 0.96, TLI = 0.94, RMSEA = 0.07, SRMR = 0.05  Ownership factor: CFI = 0.97, TLI = 0.94, RMSEA = 0.08, SRMR = 0.04  Parental Support factor: CFI = 0.99, TLI = 0.99, RMSEA = 0.03, SRMR = 0.02) | -Continuity of care: 0.622  -Collaborative relationships: 0.822  -Integration: 0.817  -Ownership: 0.757  -Prenatal support: 0.754 |  | Pearson correlations between HCTOI subscales:  Parental support vs Ownership: 0.48  Parental support vs Continuity of care: 0.37  Parental support vs Collaborative relationship: 0.52  Parental support vs Integration: 0.49  Ownership vs continuity of care: 0.58  Ownership vs Collaborative relationship: 0.67  Ownership vs Integration: 0.76  Continuity of care vs Collaborative relationship: 0.65  Continuity of care vs Integration: 0.57  Collaborative relationship vs Integration: 0.62  Pearson correlations between HCTOI subscales and the PedsQL, Diabetes Distress Scale:  -Continuity of care: 0.38  -Collaborative relationships: 0.5  -Integration: 0.55  -Ownership: 0.68  -Prenatal support: 0.36  Pearson correlations between HCTOI subscales and the Diabetes Self-Management Profile, Self-Report: -Continuity of care: 0.45  -Collaborative relationships: 0.59  -Integration: 0.57  -Ownership: 0.72  -Prenatal support: 0.49 |  |  |
| HNS-CHD: The Healthcare Needs Scale for Youth with CHD | 3 factors were extracted with the load ranging from 0.52–0.81; eigenvalue = 4.6; these three factors explained 62.1% of the total variance | -Health management subscale: 0.92  -Health policy subscale: 0.90  -Ondividual and interpersonal relationships subscale: 0.89 |  | Pearson Correlation statistic for total scale  Health needs for adolescents: 0.30 |  |  |
| I-HNS-CHD-s: Italian-Short Version of Healthcare Needs Scale for Youth with CHD | χ2 (62) = 121.38, p < 0.001; χ2/df = 1.95; RMSEA = 0.047; CFI = 0.982; TLI = 0.964; SRMR = 0.029; 4 factors explained the 77.60% of the total variance; loading were ≥ 0.629 on domain 1; ≥ 0.628 on domain 2; ≥ 0.514 on domain 3; ≥ 0.642 on domain 4 | -Total scale McDonald’sω= 0.76    Subscale:  -Healthcare education McDonald’s ω= 0.84  -Clinical support McDonald’s ω = 0.71  -Emotional support McDonald’s ω = 0.65  -Continuum of care McDonald’sω= 0.71 |  |  |  |  |
| Japanese TRAQ |  | -Cronbach’s alpha coefficients for total scale: 0.94  Subscales:  -0.84 for “Managing Medications,”  -0.80 for “Appointment Keeping,”  -0.88 for “Tracking Health Issues,”  -0.96 for “Talking with Providers.” |  |  |  |  |
| Not named; Funes et al. | KMO = 0.75; Bartlett’s test of sphericity was statistically significant (p < 0.0005); 2 factor structure; explained 48.8% of the variance; loading were ≥ 0.412 on domain 1; ≥ 0.407 on domain 2. Number of items that load on more than 1 factor unknown. | -Component 1 Cronbach’s alpha α = 0.68  -Component 2 Cronbach’s alpha α = 0.66 |  |  |  |  |
| Not named; Hodnekvam et al. | 7 factors identified; factor loading ranging from 0.45-0.93 | (1) Paediatric care doctor: 0.77  (2) Paediatric care nurse: 0.79  (3) Pediatric individualized care: 0.82  (4) Prepare for transition: 0.79  (5) Adult care doctor: 0.76  (6) Adult care nurse: 0.80  (7) Adult individualized care: 0.85 | -0.64 to 0.85, with six of the seven factors scoring 0.70 or higher  -The only exception was for the individual care provided by adult health services, which scored 0.64 | Spearmen rank correlations between subscales:  Pediatric care doctor vs three other pediatric subscales, one of the three had a correlation > 0.6, remaining were lower.  Pediatric care nurse vs 2 other pediatric subscales, one of the 2 had a correlation > 0.6, remaining one was lower.  Pediatric individualized care vs prepare for transition: 0.45  Adult care doctor vs two other adult subscales, one was >= to 0.6  Adult care nurse vs adult individualized care: 0.63 |  |  |
| RACER: Readiness for Adult Care in Rheumatology |  | -All but one domain met the criteria for internal consistency General Knowledge a = 0.63  -Medication Knowledge (0.92)  -Planning for Adult Life (0.75)  -Managing Your Health Condition (0.76)  -Speaking Up for Yourself (0.85)  -Knowing How to Get Around the Healthcare System (0.86) | -Compared at T1and T2 was ICC was 0.83 (95 % CI 0.79–0.86) | Pearson Correlation statistic  Self-Management Skills Assessment Guide: 0.73  TRANSITION-Q: 0.76 |  | The standardized response means for the RACER at 2 weeks post-baseline were trivial (0.048), small at 24 weeks (0.28) and moderate at 48 (0.62) and 72 (0.73) weeks post baseline, results which are in line with the hypotheses. As hypothesized, there was no significant increase in the RACER score at two weeks (T2) compared to baseline. |
| RISQ-T: Readiness for Independent Self-Care Questionnaire Adolescent |  | -Internal consistency: RISQ-T α=.78  -Knowledge, α RISQ-T=.51, α RISQ-P=.61  -Behavior, α RISQ-T=.62, α RISQ-P=.71  -Adolescent’s Perceived Importance α RISQ-T=.81  -Item-to-total correlations ranged from 0.19 to 0.55, with 75% of items on the RISQ-T | -The intraclass correlation coefficients (ICC) between RISQ scores, after six months, were acceptable for RISQ-T (r=.66) | Pearson Correlation statistic  RISQ-T Parent Report: 0.35  Diabetes Family Responsibility Questionnaire:  -0.34  Diabetes Management Self-Efficacy Questionnaire: 0.32  Problem Areas in Diabetes – Pediatrics Adolescent Report: -0.07  Diabetes Management Questionnaire: 0.26 |  |  |
| Self-Management Skills Checklist | 3 factors identified | -Adolescent Skills summary scale had good reliability: α=0.77  Skills Scale: α=0.77  Knowledge scale: α=0.44 |  |  |  | There was no significant difference between the first (M = 3.66, SD = 0.73) and second (M = 3.76, SD = 0.62) administration scores on the Adolescent Skills scale (t25 = 0.681, P = 0.50), indicating no meaningful change in adolescents’ perception of their disease-specific and general self-management skills. |
| STARx  Questionnaire; Nazareth et al. | 3 factors identified; loading were ≥ 0.515 on domain 1; ≥ 0.433 on domain 2; ≥ 0.513 on domain 3 | -Factor 1 (Disease knowledge) - 0.764; Factor 2 (Self-management) - 0.724; Factor 3 (Provider communication) - 0.687 |  | Moderately high Pearson correlations were found between parent and child total and subscale scores for those in the same family:  Disease Knowledge (r = 0.717)  Self- management (r = 0.596)  Provider Communication (r = 0.610)  Total Scale Score (r = 0.580) |  |  |
| STARx  Questionnaire-Chinese | χ2/df = 2.27; RMSEA = 0.052; CFI = 0.97; TLI = 0.96; SRMR = 0.066; 4 factors identified; factor loading for item ranged from 0.33 to 0.85 | Medication Management α = 0.836;  Health-Care Engagement α = 0.759;  Provider Communication α = 0.762;  Disease Knowledge α = 0.762 |  |  |  |  |
| TRAQ - Turkish version | 5 factors were identified; explaining 74% of the total variance; χ2/df of the scale was 2.49 (χ2=426.819; df=171; p=0.000); RMSEA = 0.06 | Keeping appointments: 0.85  Managing Medications: 0.89  Tracking health issues: 0.76  Managing daily activities: 0.70  Talking with providers: 0.76 | -test–retest correlation coefficients ranged between 0.79 and 0.93 (p<0.01). | Pearson Correlation statistic  Self-care agency scale r=0.57 |  |  |
| TRAQ-GV-15 | Kaiser-Meyer-Olkin (0.823); Bartlett’s-Test (p < 0.001); 3 Factors identified; loading were ≥ 0.388 on domain 1; ≥ 0.489 on domain 2; ≥ 0.485 on domain 3; accounting for 47.87% of the total variance | -Domain 1 Cronbach’s alpha=0.779  -Domain 2 Cronbach’s alpha=0.721  -Domain 3 Cronbach’s alpha=0.507 |  |  |  |  |
| TRAQ-SB; Johnson et al., 2019 | 1 factor; explaining 62% of the variance; factors loading ≥ 0.3 for all items; KMO = 0.73; Bartlett’s test of sphericity was significant (p < 0.05) | -Cronbach alpha of 0.92 |  | TRAQ overall r = 0.74; Pearson correlation coefficients varied from r = 0.68 to r = 0.74, among the 5 subscales of the TRAQ and the TRAQ-SB scale |  |  |
| Turkish version of Mind the Gap scale | The questionnaire is broken down into 2 sections MGS1 (Best care scale) and MGS2 (current care scale).  Factors loading ≥ 0.3 for all items in MGS1 and MGS2.  3 factors explaining 71% of total variance in MGS1 and MGS2.  MGS1: RMSEA = 0.04; CFI = 0.91; TLI = 0.92  MGS2: RMSEA = 0.06; CFI = 0.94; TLI = 0.91  KMO = 0.729 for total scale; Bartlett’s test of sphericity (p<0.01). | Factor 1 (Management of Environment)  - MGS1: 0.71  - MGS2: 0.70  Factor 2 (Staff Characteristics)  - MGS1: 0.87  - MGS2: 0.89  Factor 3 (Process Issues)  - MGS1: 0.89  - MGS2: 0.86  Total chronbach alpha values for MGS1 and MGS2 were 0.89 and 0.87 respectively. | -The test–retest correlation coefficients for adolescent across the total were 0.88.  For MGS1 the test re-test reliability ranged from 0.45-0.89.  For MGS2 the test retest reliability ranged from 0.51-0.84. | Turkish Patient Assessment of Chronic Illness Care: Pearson correlation coefficients was 0.60 |  |  |
